# Mental Health Recovery: Evaluation of a Recovery-Oriented Training Program

**DOI:** 10.1100/2012/820846

**Published:** 2012-12-23

**Authors:** G. K. M. L. Wilrycx, M. A. Croon, A. H. S. van den Broek, Ch. van Nieuwenhuizen

**Affiliations:** ^1^GGz Breburg, Institute of Mental Health Care, Scientific Center for Care & Welfare (Tranzo), Faculty of Social and Behavioural Sciences, Tilburg University, P.O. Box 90153, 5000 LE Tilburg, The Netherlands; ^2^Department of Methodology and Statistics, Tilburg School of Social and Behavioral Sciences, P.O. Box 90153, 5000 LE Tilburg, The Netherlands; ^3^GGz Breburg, Institute of Mental Health Care, Ericssonstraat 2a, 5121 ML Rijen, The Netherlands; ^4^Scientific Center for Care & Welfare (Tranzo), Faculty of Social and Behavioural Sciences, Tilburg University, P.O. BOX 90153, 5000 LE Tilburg, The Netherlands

## Abstract

*Aim*. This study investigates the effectiveness of a recovery-oriented training program on knowledge and attitudes of mental health care professionals towards recovery of people with serious mental illness. *Methods*. Using data from a longitudinal study of recovery, changes in knowledge and attitudes of 210 mental health care professionals towards recovery were explored using the Recovery Attitude Questionnaire and the Recovery Knowledge Inventory. The study uses a two-group multiple intervention interrupted time-series design which is a variant of the stepped-wedge trial design. A total of six measurements occasions took place. *Results*. This study shows that professionals' attitudes towards recovery from mental illness can improve with training. After two intensive recovery-oriented training sessions, mental health care professionals have a more positive attitude towards recovery in clinical practice. *Conclusion*. A recovery-oriented training program can change attitudes of mental health care professionals towards recovery of serious mental illness.

## 1. Introduction

With growing interest in the concept of recovery of patients with severe mental illness, the role of the mental healthcare system is receiving increasing attention. The National Consensus Statement on Mental Health Recovery defined recovery as “a journey of healing and transformation enabling a person with a mental health problem to live a meaningful life with the limitations of the illness, in a community of his or her choice while striving to achieve his or her full potential” [1]. Recovery in this sense is focused on personal growth, hope and autonomy [2], and learning to live with the negative consequences of the disease [3]. Recovery, in this way, is based on the client's perspective [4, 5]. It is seen as a continuing process of change [6] which is not illness focused. The main issue is how treatment can facilitate the recovery process of patients with long-term psychiatric problems, and how the relationship with the mental health consumer might impede or facilitate recovery [7–10]. Professionals can contribute to the recovery process [11–15] and are able to facilitate a recovery-promoting environment for people with serious mental disorders (e.g., [16]). However, for successful implementation of a recovery approach, mental health care professionals need to change or adapt their attitudes towards this new vision of recovery.

To change the traditional mental health care system to a more recovery-oriented one, many organisations train their professionals in the recovery concept. However, lack of knowledge and skills, organisational barriers (such as poor leadership), a change-averse culture, insufficient collegial support, and bureaucratic constraints may hinder the dissemination and implementation of innovative approaches [17]. A supportive factor for effective implementation is the use of understandable language, which promotes a more positive attitude towards the topic and increases perceived behavioural control over the implementation [18, 19]. Hence, to implement a more recovery-oriented care system, it is important to focus on the professional's belief in and understanding of recovery, and the ability to promote patient recovery [10, 11]. Moreover, professionals who have to assimilate a new recovery vision into their routine practice need to master a set of core competencies [20]. These competencies include effective communication, fostering hope, appropriate self-disclosure, and a mutual respectful partnership in treatment. Working in partnership, identifying individual needs and strengths [21], and responsible risk-taking are also capabilities that strengthen a new way of working with people with severe mental illnesses. Unfortunately, much of the evidence available today is of a narrative nature, whereas to validate a new recovery approach more empirical-based data are required [22]. 

Therefore, this study investigates the effectiveness of a recovery-oriented training program implemented in the Netherlands. To explore changes in knowledge and attitudes of mental health care professionals, a variant of the stepped-wedge trial design [23, 24] was used. 

## 2. Methods

### 2.1. Procedure

All mental healthcare workers of the department “Impact” (the department for long-term mentally ill people in Breda/Etten-Leur) were asked to participate in an educational program about recovery. All participants were verbally informed by their managers; they received an information flyer about the program, and gave informed consent before the study started. The educational program was mandatory for all professionals. Parallel with the educational program an evaluation study was conducted to assess the effects of the educational program. The management team explicitly encouraged participation in the evaluation study. 

Prior to the start, the regional Medical Ethics Approval Committee for Mental Health Care Institutions (METIGG) was approached. According to the Medical Research Involving Human Subjects Act (WMO), ethical approval was not required.

### 2.2. The Training Program

In order to implement the new recovery vision and to achieve a culture change within the mental health organisation located in Breda, a recovery-oriented care project was developed by three major mental health care organisations: that is, two rehabilitation organisations (Rehabilitation '92 [25] and STORM rehabilitation [26]) and one peer-support organisation (HEE [27]). The “Recovery and recovery-oriented care” project was developed especially for the mental healthcare network “Impact” where people with chronic psychiatric disorders, for example psychotic disorders, are treated. Inpatients and outpatient's settings are involved. The main goal of the project was to create and promote a new culture towards recovery from serious mental illness: how can treatment promote the recovery process of patients with long-term psychiatric problems and does the relationship with the mental health professional facilitate recovery [13, 14, 21]? 

The educational program was given in two separate intensive training sessions, one in 2008 and a second one in 2009. The training program was developed for all professionals who are in close contact with the mental health care patients, like there are psychologists, psychiatrists, secretaries, managers, and nurses. The training program consisted out of two modules given in a two-day session every six months. The first module was focused on the basics of recovery-oriented care in order to familiarise the professional with the concept of recovery. The second module was focused on the recovery-oriented attitude of the professional. Both courses were presented by an expert by experience from a peer support centre and a professional rehabilitation teacher. A more extensive description of the development of the training program is given in Boevink et al. [28].

**Figure 1:**
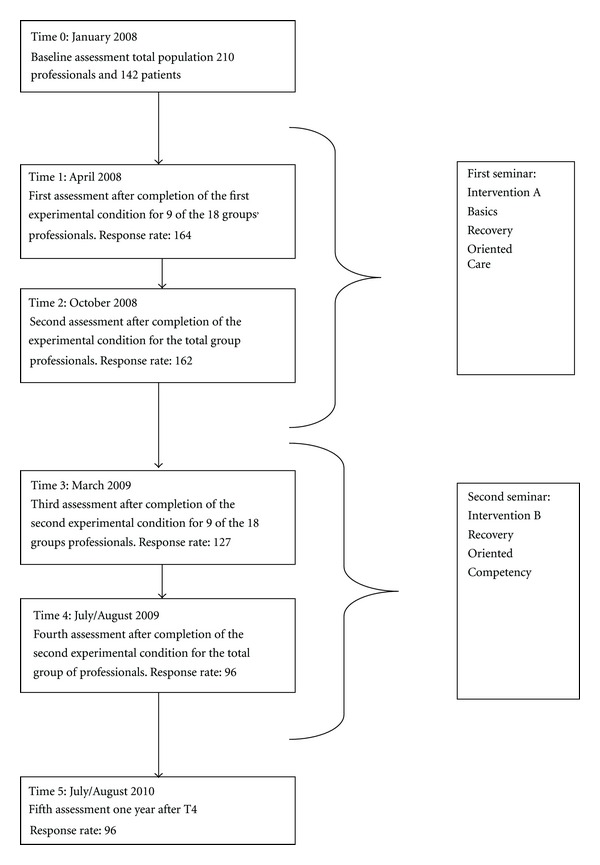
Flowchart of the training and measurements occasions.

 The participants were randomly selected and eighteen groups were formed with 10–16 professionals per group. The first module “Basics of recovery and recovery-oriented care” (intervention A) was given in the first half of 2008. The second module (intervention B) was given in spring and summer of 2009. This seminar was focused on attitude towards recovery and the way the professional is able to stimulate and facilitate recovery within the client. An overview of the training seminars (experimental conditions) with the different measurement occasions and the corresponding response rates is given in Figure 1. Both seminars were given in close cooperation with an expert by experience from the peer-support organisation. 

### 2.3. Sample of Professionals

The sample of professionals was recruited at Impact. All 270 professionals were invited to participate in this longitudinal study. Of these, 210 agreed to participate: their average age was 43.3 (range 20–60) years and 74% was female. Their mean period of employment in the mental healthcare sector was 13.2 years and their mean period of experience dealing specifically with long-term psychiatric disabilities was 11.3 years. The sample of professionals consisted of psychiatrists, psychologists, psychiatric nurses, day-activity workers, care assistants, and other professionals in close contact with clients. Two subsamples were formed which each consisted out of nine groups of 10 to 16, randomly selected, professionals. The aim of the educational program was to induce a culture change towards recovery in the entire organization. This was the rationale to include (additional) staff members, such as managers and secretaries, working in different settings.  Table 1 presents an overview of the demographic characteristics of the study group. 

*Note.* Parallel with the measurement occasions for professionals, data were collected of 142 mental health consumers for which the Mental Health Recovery Measure (MHRM; [29]) and the Recovery Promoting Relationship Scale (RPRS; [30]) were used. These data will not be used in this study. 

### 2.4. Instruments

In this study, the Dutch versions of the Recovery Knowledge Inventory (RKI; [31]) and the Recovery Attitude Questionnaire (RAQ; [32]) were used. Both instruments are self-report questionnaires for professionals. The original questionnaires were translated into Dutch using a backward-forward translation procedure [33]. Details of the translation procedure and the psychometric properties of the Dutch scales are provided in Wilrycx et al. [34]. 

#### 2.4.1. Recovery Knowledge Inventory (RKI)

The RKI was used to assess the professionals' general knowledge about recovery over time. The Dutch version of the RKI consists of 14 items and focuses on “Knowledge of recovery”. Cronbach's alpha for this total scale was 0.80. 

#### 2.4.2. Recovery Attitudes Questionnaire (RAQ)

The RAQ was used to assess the professionals' feelings and attitudes towards recovery. The Dutch version of the RAQ consists of 5 items and focuses on “Attitudes towards recovery”. Cronbach's alpha for the total scale was 0.61. 

Correlation between the RAQ and the RKI scale scores was 0.20 (*P* = 0.004); this is a significant but low enough correlation to demonstrate that both scales measure different constructs and each instrument has sufficient discriminant validity.

Both instruments were send by mail after each intervention, and participants were asked to complete and return these questionnaires within two weeks.

### 2.5. Study Design

In this study, a two-group multiple intervention interrupted time-series design was used which is a variant of the stepped-wedge trial design. The stepped-wedge trial design [23, 35, 36] is a repeated-measures design in which the sample is randomly divided into several subsamples which are observed at all time points but differ with respect to the moment at which the experimental intervention is implemented. At the first measurement occasion, all subsamples are observed prior to the intervention. The moment at which the intervention is systematically implemented varies across the subsamples, but at the end of the study all subsamples are observed after the intervention.

For the present study, the basic stepped-wedge design first was modified because two different interventions (represented by the symbols A and B; see Table 2) were implemented at different times. Intervention B always followed after intervention A. Another modification of the basic design concerned the number of subsamples that could be formed. Although in the present study six measurement occasions were planned, only two subsamples could be formed because of the way the educational program was organised. The training sessions were delivered in two sessions over two years. Table 2 shows when the two interventions were implemented in each subsample.

At the first time point (0 = baseline measurement), both subsamples were observed before implementation of either A or B. The first subsample was then observed twice after implementation of A, and three times after implementation of B. The second subsample was observed twice before intervention A, twice after intervention A, and finally twice after intervention B. In both subsamples, a total of six measurement occasions (1–5 = follow up measurements) were planned. At the end of the study, all participants had received both interventions. The time point 5 was observed one year after the time point 4. Since assignment of the subjects to the subsamples was carried out randomly, no systematic differences were expected to exist between the two subsamples.

### 2.6. Statistical Analyses

The differences between the means of the RKI and the RAQ before and after intervention were tested using a random intercept multilevel regression model with time periods nested within individuals. This model is described in a linear structural equation model and its parameters are estimated by means of AMOS. This software package allows full information maximum likelihood estimation of a model without discarding any observed score in the sample. The analysis of the data was based on the following model. Let *i* represent a participant in anyone of the subsample *c* = 1 or *c* = 2, let *t* denote measurement occasion and *y*_*cit*_ the observed score on a dependent variable for participant *i* in subsample *c* at occasion *t*. Then, the following decomposition of the individual scores was postulated:

(1) $$\begin{array}{c} y_{cit}=\mu_{ct}+\upsilon_{i}+\varepsilon_{cit}. \end{array}$$

In this expression, *μ*_*ct*_ represents the population mean for subsample *c* at measurement occasion *t*. The term *υ*_*i*_ is an individual random effect that is included in the model for capturing systematic differences between subjects in the general response level. Finally, the quantities *ε*_*cit*_ are the individual error terms. All random effects are assumed to be mutually independent. Due to the design of the stepped wedge trial design, some of the subsample means *μ*_*ct*_ are constrained to be equal (see Table 2). 

In Table 2, the symbols O_1_ and O_2_ represent the observations before the implementation of intervention A in both subsamples; the symbols A_1_ and A_2_ represent the observations after the implementation of intervention A but before implementation of B in both subsamples; finally, the symbols B_1_ and B_2_ represent the observations after implementing B.

The first hypothesis, that is whether there are no systematic differences between the means of the two subsamples, resulted in the joint test of three subhypotheses, *μ*_O1_ = *μ*_O2_, *μ*_A1_ = *μ*_A2_, and *μ*_B1_ = *μ*_B2_. When this first hypothesis cannot be rejected, the number of means to be estimated is further reduced and only three different means remain to be estimated (second hypothesis): *μ*_O_ representing the mean before any of the interventions, *μ*_A_ representing the mean after implementing A but before implementing B and, finally *μ*_B_ representing the mean after implementing B. This second hypothesis tested the following subhypotheses: whether intervention A has an effect, that is, *μ*_A_ = *μ*_O_, whether intervention B has an effect that is, *μ*_B_ = *μ*_O_, and, whether the effects of B and A are equal, that is, *μ*_B_ = *μ*_A_. In the model, the effects of intervention A and B are estimated by the differences *μ*_A_ − *μ*_O_ and *μ*_B_ − *μ*_O_, respectively.

Both hypotheses mentioned above were tested by different linear structural equation models in AMOS. The significance of the models was tested by means of conditional likelihood ratio tests which under the null hypothesis follow chi-square distributions with their degree of freedoms equal to the number of constraints imposed on the model parameters. This requires two consecutive models to be tested: in one model without imposing the constraints on the subsample means implied by the hypothesis being tested, and one in which these constraints are explicitly imposed. Because the two models are nested, the conditional chi-square test is obtained by subtracting the chi-square values of the two analyses [37].

## 3. Results

### 3.1. Results for the RKI

For the RKI, the null hypothesis that there were no systematic differences between the means of the two subsamples could not be rejected with a *χ* ^2^ = 1.641 with 3 degrees of freedom (*P* = 0.650). The sample estimates of the three means (represented by *M*_O_ = mean before intervention A, *M*_A_ = mean after intervention A but before intervention B, and *M*_B_ = mean after intervention B, resp.,) to be estimated under the reduced model and their standard errors are

- *M*_O_ = 3.027  (0.021),
- *M*_A_ = 3.113  (0.019), and
- *M*_B_ = 3.066  (0.022).

Intervention A has a significant effect since the null hypothesis *μ*_A_ = *μ*_O_ has to be rejected with a *χ* ^2^ = 17.888 with 1 degree of freedom (*P* = 0.000). However, the null hypothesis *μ*_B_ = *μ*_O_ cannot be rejected (*χ* ^2^ = 2.939, df = 1, *P* = 0.086), and intervention B fails to have an effect. Moreover, since the hypothesis *μ*_B_ = *μ*_A_ is also rejected (*χ* ^2^ = 5.783, df = 2, *P* = 0.016), the mean after intervention B drops back to the initial level. Intervention B then seems to annihilate the positive effect of intervention A. 

### 3.2. Results for the RAQ

For the RAQ, the null hypothesis that there were no systematic differences between the means of the two subsamples could not be rejected with a *χ* ^2^ = 0.890 with 3 degrees of freedom (*P* = 0.828). The estimates of the three means to be estimated under the reduced model and their standard errors are

- *M*_O_ = 3.008  (0.029),
- *M*_A_ = 3.100  (0.031), and
- *M*_B_ = 3.176  (0.028).

Intervention A has a significant effect since the null hypothesis *μ*_A_ = *μ*_O_ has to be rejected with a *χ* ^2^ = 8.097 with 1 degree of freedom (*P* = 0.004). Also the null hypothesis *μ*_B_ = *μ*_O_ has to be rejected (*χ* ^2^ = 29.603, df = 1, *P* = 0.000), indicating that intervention B has an effect. Finally, also the hypothesis *μ*_B_ = *μ*_A_ is rejected (*χ* ^2^ = 5.783, df = 2, *P* = 0.016), and intervention B is seen to have a larger effect than intervention A.

## 4. Discussion 

This study evaluated a recovery training program for professionals in the Netherlands. Specifically, the changes in knowledge and attitudes of mental health care professionals towards recovery of mentally ill patients were investigated using a modified stepped-wedge trial design. The results suggest that over the total course of the training program, expected changes were found in attitudes towards recovery. Similar findings were reported by Crowe et al. [12], and Cleary and Dowling [11], who found that mental health professionals had more favourable beliefs and more positive attitudes related to recovery during the course of the training program. One explanation for the positive results in the present study might be the way the intervention was given. The trainer was an expert by experience, who reflected on the quality of treatment received in the past thereby generating self-reflection. According to Bandura [38] self-reflection can result in a change of attitudes. Because the professional undergoing training was confronted with reports of maltreatment stories, the educational program had an emotional as well as a learning impact. Secondly, the use of understandable/appropriate language might contribute to the positive effect and the perceived behavioural control over the implementation. 

Positive results were also found for the change in knowledge after intervention A on knowledge about recovery. However, intervention B (that focused mainly on attitude) had a negative effect on knowledge rather than the expected positive cumulative result. This negative result towards knowledge of recovery might be explained as follows. First, the program developers and the department managers did not investigate the professionals' readiness to change. Before educating or training people, it is important that professionals are motivated to learn [39, 40]. Second, the lack of rehearsal of knowledge about recovery during intervention A might be responsible for the negative results after intervention B. Studies show that rehearsal is crucial for the implementation of information and is essential for the integration of new knowledge in long-term memory [41–43]. Third, the relatively high age of the professionals might play a role in this poor result, since younger and less experienced people are generally more eager to learn [16]. Forth, because the course was mandatory the extrinsic motivation to change might have been greater than the intrinsic motivation to learn [40]. Finally, as we now know from the recently developed Refocus model [44], the implementation of recovery is much more complex than how it was offered in the training program for professionals discussed in this study. The training program was based on just one part of the Refocus implementation model, namely, staff values, knowledge and partnership and lacked specific training at the work place. 

### 4.1. Limitation and Strengths

This study has a number of limitations. First, the original stepped-wedge trial design needed a modification because of the way the training program was organised. Epidemiological studies using this design have generally explored the long-term effect of just one intervention [44–46], whereas in the present study, the effects of two interventions over a two-year period were examined. Second, there are no reference data for comparison purposes. Reference data of epidemiological studies are available, but data from psychosocial studies using this two-group multiple intervention interrupted time-series design are lacking. Third, the multiple measurement occasions made the research vulnerable; because six measurements took place this made it difficult to maintain the cooperation/motivation of the professionals. 

The specific strength of this study is that it has many advantages: it enables to investigate the stepwise implementation of new ideas over time, in a practical situation that does not permit to deliver the intervention simultaneously to all participants [20]. Because of the stepwise implementation of the new recovery concept, professionals could maintain their routine practice. Another strong point is that subjects were randomly assigned to one of the two subsamples defined in the modified stepped-wedge trial design. The fact that no systematic differences were found between the two subsamples demonstrated that the randomization was successful. Finally, the modified stepped-wedge trial design is a within-subject design, which makes the inclusion of a “no intervention” control group less urgent.

## 5. Conclusion

The study shows that staff knowledge and attitudes regarding recovery from mental illness can improve with training. Mental health care workers have more positive attitudes towards recovery in clinical practice after completing the two training sessions. Furthermore, the modification of the stepped-wedge trial design—which resulted in a two-group multiple intervention interrupted time-series design—has proved to be a useful and promising design to investigate different groups of subjects within behavioural science. More research is needed about the use and the advantages of this specific design within behavioural science. More follow-up research is necessary to investigate how to stabilize changes in attitudes of professionals over time and to evaluate the effectiveness of the recovery-oriented training programs within the mental care.

## Figures and Tables

**Table 1:** Demographic characteristics of the study group (*n* = 210).

	*N*/mean	%
Female	157	74
Mean age in years (SD)	43.3 (10.8)	
Mean working history, in years (SD)	13.2 (10.2)	
Mean working history within chronic care, in years (SD)	11.3 (9.5)	
Working discipline		
Psychiatrist/psychologists	6	3
Psychiatric nurse	117	56
Occupational therapist	32	15
Placement supporter	11	5
Case manager	10	5
Care assistant	10	5
General staff members of Impact*	24	12
Setting of employment		
Clinical intensive care	39	19
Crisis intervention team	6	3
Sheltered and protected care	65	31
Ambulatory care	12	5
Day-activity centre	42	20
Impact general*	26	12
Information not available	20	10

*The Impact general group includes managers, secretaries, administrative employees, and a priest.

**Table 2:** Schedule for the two subsamples in the present study and hypothesized equality of means in the analysis.

	Time					
	0	1	2	3	4	5
Subsample 1	O_1_	A_1_	A_1_	B_1_	B_1_	B_1_
Subsample 2	O_2_	O_2_	A_2_	A_2_	B_2_	B_2_

Note: The subscripts indicate the means that are constrained in the analysis.
